# Periscope

**Published:** 1888-03

**Authors:** 


### PERISCOPE.

### MEDICINE.

**Tuberculosis in Infants—Hæmoptysis.**—Dr. P Mantel makes a valuable contribution to the study of the conditions under which hæmoptysis occurs in children under the age of seven years. From three cases under his own care, and from the published reports of twenty-seven other cases, he draws the following conclusions:—

1. Tuberculosis in children under seven years may, as in adults, be accompanied by hæmoptysis, which may be either intra-pulmonary or extra-pulmonary in origin. In the latter case the cause of hæmorrhage is generally an ulceration of the pulmonary artery or one of the other large vessels in the mediastinum starting from a suppurating tubercular mediastinal gland. He confines himself to the consideration of intra-pulmonary hæmoptysis, which is less common in infants than in adults, while the contrary holds for extra-pulmonary.

2. Intra-pulmonary hæmoptysis occurs less often in children than in adults, because, as a rule, the vessels are not degenerated. When these hæmorrhages do occur in infants, in most cases they are the victims of hereditary syphilis as well as tuberculosis; syphilis producing disease of the vessels.

3. The mechanism appears to be as follows: Syphilis and tuberculosis, by vitiating the blood, predispose to coagulation occurring in the vessels; by pressure of enlarged glands on the pulmonary artery, and hindrance to the respiratory movements, a thrombus forms in the pulmonary artery; this causes a passive congestion in the related parts of the lung, and the vessels in it, already weakened by syphilis, give way.

4. The lesions found *post mortem* are of especial interest in two points: first, the impossibility, in most cases, of finding the precise source of the hæmorrhage; and, secondly, the constant presence in the stomach of a quantity of altered blood. The clinical symptoms resemble those of hæmatemesis, and the characters of the blood vomited correspond to this. The blood appears to flow slowly from the lungs, to be swallowed, and then, when the stomach is distended, to be suddenly vomited through the mouth and nose. In some cases, where the hæmoptysis takes place still more gradually and is latent, the author thinks that it may be suspected by the occurrence of melæna for several days.—*Le Progrès médical*, December 10th, 1887.

**Cardiocentesis; or, Aspiration of the Cardiac Chambers, more especially of those on the Right side, by M. I. Bruhl.**—First a number of recorded cases are alluded to, in which the heart was accidentally punctured. They are chiefly cases in which pericardial effusion was diagnosed, but in which the actual lesion was found to be cardiac dilatation: the accidental puncture was made, in most of the cases, into the cavities of the right side of the heart. Since no ill-effects followed the accident in these instances, cardiocentesis may be considered as a practicable operation, though not to be attempted except when it appears that there is no other effective mode of treatment.

The indications for cardiocentesis are—an over-distended right heart, without any organic affection of the valves; the systole being at the same time weak, and the peripheral vessels empty. All these conditions must exist together, and to be successful the operation must be done early in the progress of the case. In presence of the above symptoms, bleeding from the veins of the arm would be useless. The author enters fully into the difficulties of the diagnosis, which consist in the determining with certainty the absence of valvular lesion, and of pericardial effusion.

The method of performing cardiocentesis is simple. The needle should be a small capillary trocar, with cannula, about 1½ mm. in diameter. After the thoracic wall has been traversed, it must be pushed into the heart with one movement, with the patient in a position midway between lying and sitting: the amount of blood to be removed varies with the case, bearing in mind that blood removed directly from the heart represents in effect six or eight times that removed from the arm in an ordinary bleeding. Either the right auricle or the right ventricle may be punctured. The former may be reached through the third or the fourth right intercostal space; the puncture must be made close to the sternum, to avoid the internal mammary artery and vein. The third space is to be preferred, because it is larger; and there is danger, if the fourth space be selected, of wounding the tricuspid valve, the coronary artery, or the intra-cardiac nerve-ganglia.

The ventricle may be reached by a puncture made in the fifth left intercostal space, three centimetres from the left sternal border. One must remember, that in animals pricking of a spot situated at the lower part of the superior third of the ventricular septum, at a distance of one centimetre below the anterior branch of the coronary artery, determines instant and permanent arrest of the ventricular contractions. This spot probably exists in man, although we have no positive knowledge of its exact situation.

Steiner says that a puncture of the ventricle is devoid of pain or danger, but that the needle must be lightly held to allow it to follow freely the movements of the heart, in order to avoid tearing the myocardium.

*Complications.*—Wound of the coronary artery, only possible when the puncture is made in the fourth right space, led in one case to rapid death from bleeding into the pericardium.

Wound of the aorta has happened twice—once to Westbrook, in whose case the aorta was pricked but not penetrated; great pain was at once felt. The second instance was in a case under the care of Dr. Shingleton Smith (reported in this Journal, 1885, p. 189); the puncture was made by Mr. Dacre. The author points out that no benefit is to be looked for from removal of blood from the aorta, and that the accident may be avoided by keeping the needle during puncture exactly in the antero-posterior direction.

Wounding of the vena cava, pulmonary vessels, and nerve-ganglia have never occurred.

Tearing of the myocardium leads to death from bleeding into the pericardial sac. If the needle is allowed to follow the cardiac movements, it is only likely to happen when the heart-muscle is in a state of fatty degeneration; and should there be any evidence of such a condition, the operation is contra-indicated.

Lastly, tearing of the posterior wall of the punctured cavity causes immediate death. Since the cavities vary in depth, this accident can only be avoided by taking care not to introduce the needle to a depth exceeding four to five centimetres.

The author concludes that—

1. Cardiocentesis is a practicable operation, not dangerous in the majority of cases. It is most applicable to cases of dilatation of the right heart, without any organic lesion.

2. Either the right auricle or ventricle may be punctured at the sites mentioned above; the use of the aspirator renders the operation more easy.

3. Puncture of the right ventricle is to be preferred, because the greater thickness of its walls gives less likelihood of tearing the muscle, and so bringing about hæmo-pericardium; and because the auricle acts rather as a reservoir than as a propeller of the blood, and therefore emptying the auricle does not much diminish the work of the heart.

4. Cardiocentesis acts both by mechanically exciting the heart and by relieving it of blood.

5. More experience is necessary to pronounce definitely on the operation, the object of the paper being to show that it is possible, and not to be rejected without consideration.—*Le Progrès médical*, December, 1887.

**Antipyrin.**—Dr. Pampoukis, of Athens, in common with other medical practitioners in Greece, has found antipyrin exceedingly efficacious against the indigenous malarial fevers. It was at first given to patients who found no benefit from quinine, either from idiosyncrasy, or from its effects having worn off by repeated use. Found successful in these, antipyrin was given to other patients with unvarying success. Their ages varied from five to forty years. The temperature after absorption of the drug became normal or subnormal. Even when given during the rigor, it stopped the further progress of the paroxysm.

Antifebrin seems equally efficacious. It acts by producing profuse sweating, and brings the temperature down within an hour after its administration.

Antipyrin is preferred by the author to antifebrin, because it seems to be the safer remedy.

In a paper by M. Robin, on the influence of antipyrin on tissue-change, the following conclusions are arrived at:

1. Antipyrin diminishes both the quantity of the urine, and also certain solids; urea and total nitrogenous excreta, chlorides, phosphoric and sulphuric acid, and sulphates.

2. It increases the excretion of uric acid; and the amount of incompletely oxidised sulphur and phosphorus.

3. Its most important action is the relief of pain. In common with other analgesics, the relief of pain is accompanied by an increased excretion of incompletely oxidised phosphorus, showing that the metabolism of nervous tissue is modified, and its excitability thereby lessened.

4. It diminishes the processes of oxidation and disintegration in the tissues generally, at the same time replacing part of the urea, often secreted in excessive amount, by uric acid and other nitrogenous extractives less easily eliminated than urea. Judging from the action of other depressants of nervous activity, the effect of antipyrin on the nervous system is dependent on this modification of general tissue-change.

5. Antipyrin has some antiseptic power. M. Robin proposes to re-name it analgesin, because he considers it of little value as an antipyretic, but useful as an analgesic.—*Le Progrès médical*, January 7th, 1888; *Gazette des hôpitaux*, December 8th, 1887. J. Michell Clarke, M.B.

### SURGERY.

**Discussion on Intestinal Obstruction.**—Dr. Lewis A. Stimson of New York read a paper on this subject before the Medical Society of the State of New York.

The principal points of diagnosis and treatment suggested by him and those who joined in the discussion are these:

In 50 per cent. of the cases of acute or subacute intussusception, a tumour can be felt in the abdomen. The less acute the case, the more difficult the diagnosis. In stricture there may be no symptoms previous to the acute attack which causes the obstruction: this, however, is exceptional. Stercoraceous vomiting is not always a sign that the matter comes from the large intestine; the contents of the ileum acquire a fæcal odour. Among cases in which spontaneous relief sometimes occurs are intussusception and stricture of the large intestine, both of which have peculiar symptoms and are not impossible of diagnosis.

Surgery can save rather over 30 per cent. of these cases. Desperate cases do recover, with or without operation; but if the symptoms do not improve very early, an operation is indicated. The only way to be able to make an early and exact diagnosis is to carefully examine every case of ordinary colic, even in its early stage. Palpation then made will often discover a tumour in the abdomen. Tympanites soon develops, and then operation is difficult or impossible. No time should be lost in endeavouring to make an exact diagnosis. The only important point is to discover whether or not obstruction really exists.

Spinach-green vomited material is not proof of the existence of peritonitis.

If obstinate constipation, pain, and vomiting have existed for forty-eight hours, laparotomy should be at once performed.

Enterostomy is indicated to relieve urgent symptoms, and whenever the diagnosis is uncertain or impossible, and whenever tympanites is very great. Simple puncture of the gut to relieve tympanites is dangerous, as the openings do not close, on account of the distension and paralysis of the bowel. Laparotomy is indicated when the diagnosis of a curable cause is certain; when a small tumour can be felt in the abdomen; when a neoplasm is present which admits of resection; and when peritonitis is present and requires drainage of the peritoneum. Chloroform is considered better than ether as an anæsthetic during the operation, and the thorough narcosis produced by morphine prior to operation diminishes the shock. The early operator is the man who will have the best results.—*Medical Record*, Feb. 11th, 1888.

**Congenital Club-foot cured by Excision of portions of the Tarsus.**—Dr. H. B. Sands brought two of these cases before the New York Surgical Society.

Case I.—Martha H., aged twelve, had a very exaggerated talipes equino-varus, with marked supination. She walked on the dorsum and outer side of the foot, where a large bursa had developed. To overcome the deformity, a wedge-shaped piece of bone was removed from the outer side of the foot. This included portions of the os calcis and cuboid, and the joint between them. As this was not sufficient to overcome the deformity, the rest of the cuboid and the external cuneiform bone were next excised. The deformity still persisting, it was found necessary to remove portions of the middle and internal cuneiform and part of the scaphoid. The abnormal rotation was then overcome, but it was still necessary to divide the tendo Achillis: this allowed the foot to be held in almost perfect position; slight plantar flexion, however, remaining. A plaster-of-Paris splint was immediately applied. Two months afterwards the foot was in good position, with the exception of a slight talipes equinus—a not uncommon complication. Dr. Sands, however, expected to overcome this by the usual orthopædic treatment. There was a considerable amount of movement at the ankle-joint.

Case II.—Sadie S., aged six. The foot in this case was in much the same condition as that of Martha H. A different plan of treatment was, however, adopted.

An incision, two and a half inches long, was made on the outer part of the dorsum extending backward, just below the external malleolus. The astragalus was first removed, but this had little effect on the deformity. The anterior extremity of the os calcis and a part of the cuboid were then removed; and after excision of a portion of the external malleolus, three-eighths of an inch in length, and division of the tendo Achillis, a perfect position was obtained. The foot was dressed and put up in plaster-of-Paris. At the end of four weeks this was removed, and the wound found to be healed. The splint was reapplied and removed again three weeks afterwards, when the patient was discharged. At that time there was good movement at the ankle, but Dr. Sands could not say whether this would remain. Excision of the astragalus is supposed to correct the deformity better than when a wedge-shaped piece of the tarsus is removed. An objection to the latter plan is that the foot is shortened, and the arch destroyed or weakened. This does not happen when the astragalus alone is removed. When this is done, the shortening of the leg tends to overcome the plantar flexion; so that it is sometimes found unnecessary to divide the tendo Achillis in order to correct this part of the deformity.—*New York Medical Journal*, Feb. 11th, 1888.

**Sudden Death after a Blow on the Testicle.**—Ivanoff (*Bulgarische Med. Spir.*) records the following case:

A middle-aged man was engaged in an altercation with a woman in the street, when she struck him a violent blow on the scrotum. He sank at once unconscious, and died in a few minutes before the surgeon could arrive.

At the autopsy nothing abnormal, except slight hyperæmia of the brain, was found to account for death. Ivanoff considered this to have resulted from syncope, due to the excessive pain caused by the blow on the testicle.—*New York Medical Journal*, Feb. 11th, 1888.

**Examination of Urethral Discharges.**—In case of doubt as to the exact nature of an urethral discharge, a little should be placed on a glass slip, and examined under the microscope, with or without the application of a cover-glass. The presence of spermatozoids in any quantity will be very certain indication that the flow is true spermatorrhœa, and their absence is almost certain proof that it is due to urethral inflammation or prostatorrhœa.

If the matter under examination shows the following morphological elements, it is indicative of urethral inflammation, viz.; leucocytes in great abundance, cylindrical epithelium, and red blood-corpuscles. In prostatorrhœa the fluid will contain little whitish masses of cells, mostly cylindrical, humourous, highly refractive, colourless granules, of a size varying from one-tenth to one-half that of a red blood-corpuscle, and a certain number of small starch-like masses of various shapes and sizes.—*St. Louis Medical and Surgical Journal*, December, 1887.

**The Connection between Head Injuries and Inflammation of the Lungs.**—Roche (*Vjrsche. f. Gerichte Med.*, N.F. XLVII., i., p. 12, 1887) has classified the lung affections as follows:

A. Those accidentally present with the injury, such as the so-called fibrinous contusion pneumonia, hypostatic pneumonia, and metastatic pneumonia.

B. Those which occur solely in consequence of the cranial injury—deglutition pneumonia, and that caused by the inspiration of foreign bodies, which occur especially in consequence of simultaneous paralysis of the cortex and the vagus centre, or by the first simply through unconsciousness.

Roche summarises his conclusions as follows:

1. Proof of the causal connection of injury to the head and fibrinous pneumonia cannot be produced.

2. Hypostatic pneumonia complicating cranial injury, presupposes great prostration of the person injured. This can have occurred before the injury (old age), or be caused by it.

3. Metastatic pneumonias following injuries of the head always indicate an infection of the wound. They therefore stand in direct connection with the lesions.

4. Deglutition, foreign body, or vagus pneumonias which develop after cranial lesions, stand in the most direct connection with the latter. They indicate, relatively, intense application of force, and are to be looked on as the consequences of paralysing effects on certain cerebral lesions.—*Cincinnati Lancet-Clinic*, Jan. 21st, 1888.

W. J. PENNY.

### LARYNGOLOGY.

**Treatment of Nodules on the Vocal Cords.**—Wagnier treats these little nodules, which are usually the result of a preceding catarrhal laryngitis, and which give rise to so much interference with the clear speaking and singing voice, by means of astringents in pigment or powder form, and usually cures them by these means. If they resist astringents, then caustics, such as solid nitrate of silver, must be applied; and for the more voluminous growths, chromic acid or electro-cautery.—*Revue mensuelle de Laryngologie*, February, 1888. [I have found these proliferations where they are, as is often the case, growing from a considerable length of the vocal cord (chorditis tuberosa of Türck), yield after repeated painting of the parts with solution of chloride of zinc, perchloride of iron, or nitrate of silver. Lactic acid, in 50 or 60 per cent. solution, also is valuable.]

**Infectious Otitis.**—Hessler describes 17 cases in which, as the result of preceding excoriation, micro-organisms found their way into the tissues of the ear. The symptoms were rigors, fever, and much swelling in and around the ear. It does not lead to secretion of pus, and so can be distinguished from furuncle. Treatment: Local blood-letting, hot and cold bathing, and incision, are often useless. On the other hand, the use of solutions of corrosive sublimate locally is curative. —*Revue mensuelle de Laryngologie*, February, 1888.

BARCLAY J. BARON, M.B.

